# Are There Gender-Dependent Study Habits of Medical Students in Times of the World Wide Web?

**DOI:** 10.1155/2018/3196869

**Published:** 2018-12-06

**Authors:** A. Benditz, L. Pulido, T. Renkawitz, T. Schwarz, J. Grifka, M. Weber

**Affiliations:** ^1^Department of Orthopaedics, University Medical Centre Regensburg, Asklepios Klinikum Bad Abbach, Kaiser-Karl-V-Allee 3, 93077 Bad Abbach, Germany; ^2^Department of Pediatric Orthopaedics and Traumatology, Hospital Base San José Osorno, Avda Dr. Guillermo Bühler 1765, Osorno, Chile

## Abstract

This study evaluates how medical students rate the different types of teaching materials and methods available as well as possible gender-specific differences in the use of such materials. In this descriptive, cross-sectional study a questionnaire with short, one-dimensional questions with a 4-step Likert scale was developed by a presurvey within 493 students (4th year) at a University Medical School (January-December 2015). The anonymous survey was performed from July 2016 to February 2017 with 252 students within an orthopaedic surgery course at University Medical School. After exclusion of (1) nonnative speakers and (2) incomplete forms, 233 samples were included. Practical education was regarded as the most important (n=160/68.7%) teaching method followed by Internet research (n=147/63.1%) as the most important teaching material, while traditional frontal teaching (n=19/8.2%) and e-books (n=11/4.7%) ranked last. The evaluation of gender-specific differences in the use of teaching materials showed that female students prefer to highlight text (p<0.0001) as well as a trend to Internet research (p=0.053) and small-group teaching (p=0.057). Despite some gender-specific differences, traditional learning methods retain their importance besides new learning possibilities such as Internet research.

## 1. Background

Reliable medical care and medical progress must be based on solid, fundamental education. However, students' demands are increasing, and learning possibilities and sources have changed in times of the World Wide Web. Over the past 50 years, medical knowledge has increased much more than in the 500 years before [1]. Therefore, existing academic structures and educational methods must be adapted to satisfy modern needs. Furthermore, teachers and students have to continuously remain up-to-date to be able to transfer such vast amount of knowledge in a timely manner. The most challenging task is to choose a method that helps to understand and remember what has been learned. Many medical schools have already responded to these needs by adopting the latest recommended learning techniques including a shift from teacher-centred classical frontal teaching to student-centred problem-based learning [2].

In 1990, the term ‘learning style' was first described by Dunn et al. as different ways of learning and reproduction of knowledge unique for every individual [3].

Even before this definition until today, it had been discussed for decades that, on the one hand, teachers benefit from knowing about the study habits of their students so that they may adapt their teaching methods; on the other hand, students themselves should know about their individually preferred learning style [4, 5].

Another important aspect in modern education is the different study habits of female and male students. Other studies have shown differences in learning styles between men and women that influence the academic performance of medical students [6, 7].

The proportion of female students in medical schools has been growing continuously. In 2015, 89 998 medical students were enrolled at German universities in comparison to 82 333 in 1998. However, the percentage of female students in 1998 was 50% compared to 60.7% in 2015 [8].

The orthopaedic curriculum, evaluated in this study, offers a wide range of learning methods including problem-based learning, bedside teaching, small-group exercises, and extra lessons additional to traditional frontal teaching. In addition, an e-book as well as online tutorials is available.

Many questionnaires have been developed to evaluate the different learning styles. Most studies on learning styles over the past few years have been evaluated by means of the VARK-questionnaire [9–13], which is divided into 4 abstract sections: visual (V), aural/auditory (A), read/write (R), and kinesthetic (K) [14].

To easily access the study habits of the students and learn about their demands on this specific evaluated education without any differentiation in the quality of those items, a new questionnaire was developed in this study. As mentioned before, because of the increasing proportion of female students in medical schools, it was also hypothesized about possible differences in learning styles and learning approaches of male and female students.

Such gender-specific differences should be incorporated into the curriculum to suit the habits of our students, both male and female.

### 1.1. Aim of the Study

The aim of this study was to evaluate how medical students rate the different types of teaching materials available as well as the proof the hypothesis of gender-specific differences in the use of such materials.

## 2. Methods

A descriptive, cross-sectional study design was used. The study was approved by the Ethics Committee of the University of Regensburg (10-101-0122).

In a presurvey, 493 4th-year medical students (January-December 2015) were asked to define their most important item with regard to learning. Using free text, students could name a special type of learning material, a particular method, or just what they defined as most important for their individual learning style. The questionnaire for the main survey was based on the 10 most frequently mentioned items in the presurvey.

The questions were short, one-dimensional, and neutral. The questionnaire also included open questions on age, gender, and any previous jobs or academic degrees.

The questionnaire was evaluated by means of a 4-step Likert scale (‘unimportant', ‘rather unimportant', ‘important', and ‘very important'). At the end, the medical students had to rate their subjective priority of learning method by means of the following 7 items on an ordinal scale: ‘learning in small groups', ‘traditional book', ‘e-learning', ‘manuscripts online', ‘videos', ‘e-book', and ‘audio book'. The students ranked their priorities from 1 to 7 (1 = most important to 7 = not important at all). Besides, the questionnaire collected anonymized data of gender, age, social background, and their parents' profession.

In a pretest, 10 independent students had evaluated the time it took to answer the questionnaire, its structure, and intelligibility as well as its choice of scale [15].

All 252 4th-year medical students of the orthopaedic surgery course at the University of Regensburg Medical School (July 2016 to February 2017) were asked by the authors to take part in the anonymous survey. The questionnaires were distributed in both compulsory and optional lectures so that there was a fair chance to reach every student of the semester. Thus, the choice of students was not followed by a rule. Exclusion criteria were (1) nonnative speakers (n=7) to address problems in understanding and (2) incomplete forms (n=12) thus 233 samples were included in the final analysis.

### 2.1. Statistical Methods

Statistical analysis was done descriptively, reporting the relative frequency of the 4-step Likert scale in relation to the 10 items of the questionnaire of all 233 medical students. Bar charts were used for visual presentation. Ordinal scaled rankings of students' teaching priorities were analysed, reporting mean and standard deviation, and correlated with the sex of the students and their parents' profession. Mann-Whitney* U* tests were used for statistical testing. The corresponding significance level was adjusted according to Bonferroni. IBM SPSS Statistics 22 was used for statistical analysis.

## 3. Results

233 students halfway through their years of study (7th and 8th semester, 4th year) were included in the study. The ratio between female and male students was 144 (62%) to 89 (38%). An overview of the data of the study group is given in Table 1.

Practical education was regarded as the most important (n=160/68.7%) teaching method followed by Internet research (n=147/63.1%) as the most important (n=160/68.7%) teaching material, while traditional frontal teaching (n=19/8.2%) and e-books (n=11/4.7%) ranked last. The evaluation of gender-specific differences in the use of teaching materials showed that female students prefer to highlight text (p<0.0001) as well as a trend to Internet research (p=0.053) and small-group teaching (p=0.057).

### 3.1. Main Findings

**Figure 1:**
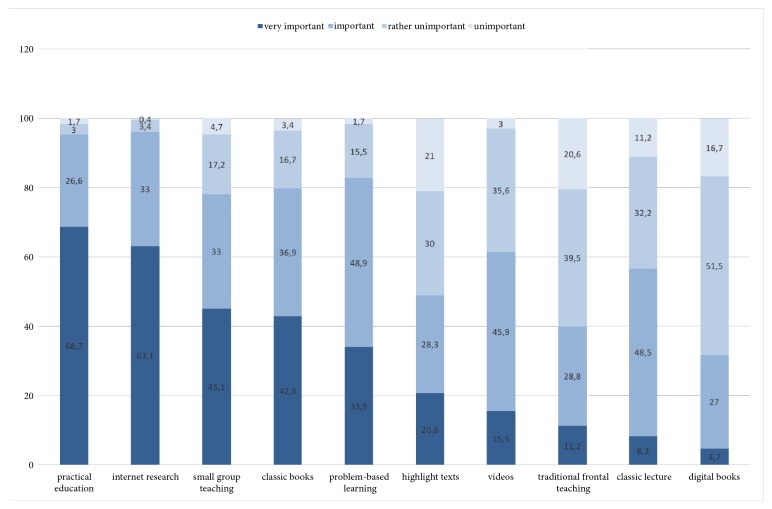
Bar charts of percentage rated on a Likert scale (1-4), sorted by highest percentage for ‘most important'.

Practical education was regarded as the most important (4 on the Likert scale) teaching method by 160 (68.7%) students followed by Internet research 147 (63.1%) as the most important teaching material. Small-group teaching was viewed as important by 105 (45.1%) students. Traditional frontal teaching (19/8.2%) and e-books (11/4.7%) were considered least important (Figure 1). The combined rating of very important and important (3 and 4 on the Likert scale) showed only small changes in the ranking. These results can also be seen when analysing the average of the 4-point Likert scale, while practical education ranked first with 3.62 (SD ± 0.632) and traditional frontal teaching was not seen to be so important with 2.54 (SD ± 0.799).

### 3.2. Group-Specific Differences

The subgroup analysis showed some differences. Due to the Bonferroni adjustment, only tendencies can be seen. Students who had not studied another subject before rated traditional frontal teaching higher (2.56, SD ± 0.81) than students who went to medical school for a second degree (2.00, SD ± 0.79, p=0.038). Students who had participated in vocational training prior to medical school rated classic books with 3.41 (SD ± 0.77) higher than students without any previous work experiences (2.12, SD ± 0.84, p=0.019). Also, videos 3.02 (SD ± 0.70) and 2.64 (SD ± 0.74) were rated higher by this group (p=0.001). No differences were found between students with and without parents who were medical doctors themselves.

### 3.3. Gender-Specific Differences

Some differences were found between the needs of female and male students. Female students preferred to highlight text (2.72, SD ± 0.93 to 2.10, SD ± 0.84, p<0.0001) as well as Internet research (3.65, SD ± 0.88 to 3.28, SD ± 0.62, p=0.053) and small-group teaching (3.26, SD ± 0.87 to 3.06, SD ± 0.62, p=0.057), while male students preferred digital books (2.36, SD ± 0.80 to 2.10, SD ± 0.73, p=0.038).

### 3.4. Ranking Priority

**Figure 2:**
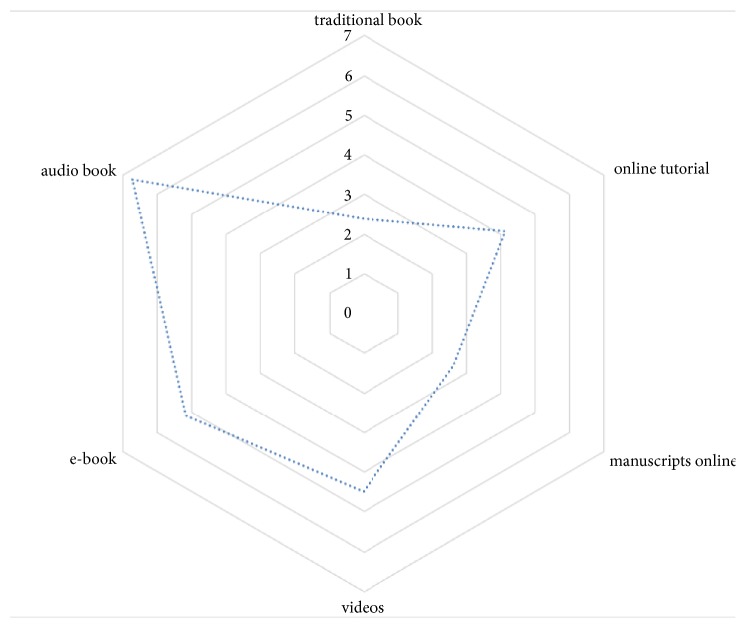
Rating of students' subjective priority of learning within 7 items on an ordinal scale. The students ranked their priorities from 1 to 7 (1 = most important to 7 = not important at all).

Analysis of the priority list showed that not only new media such as online tutorials (4.1, SD ± 0.21) or online manuscripts (2.6, SD ± 0.31) but also classic books ranked first (2.4, SD ± 0.22), while e-books (5.2, SD ± 0.44) and audio books (6.7, SD ± 0.37) ranked last. (Figure 2)

## 4. Discussion

The aim of this study was to evaluate how medical students rate the different types of teaching materials available as well as possible gender-specific differences in the use of such materials. In the group of 233 students of the 4th year of medical school, the ratio of female to male students was 62% to 38%, which corresponded to the ratio of female to male students throughout Germany in 2015 (54 638 (60.7%) female students) [8].

Several studies have shown that learning style preferences are not related to a student's academic achievements [9, 10]. In addition, most studies on learning styles over the past few years have been evaluated by means of the VARK-questionnaire [9–13, 16, 17]. The studies have shown that students prefer multimodal learning styles but on abstract sections: visual (V), aural/auditory (A), read/write (R), and kinesthetic (K) [14].

This study is the first in which the 10 most important items related to study success were selected by the students themselves. Although learning style preferences are not related to a student's academic achievements, students benefit from teachers who know about the importance of different learning methods or materials.

Discussing the presurvey, each of the 10 items evaluated by means of our questionnaire plays an important role for students; the results also showed a rating within these 10 items. The students chose traditional items such as frontal teaching, classic books, and teaching in small groups but also modern possibilities, for instance, Internet research and e-books.

The item most important for the entire group was practical education with 68.7%, which was rated 4 (most important). Particularly, in orthopaedic studies, practical exercises and bedside teaching are seen as very important for learning how to examine a patient [18]. Students ranked Internet research as second most important item (63.1%) because it enables quick and easy access to information. Many medical information sites can be used for free, and students use them, for instance, during practical instruction, to quickly obtain information on their mobile phones [19–21].

Although many studies have described traditional frontal teaching as the most effective way to transfer knowledge [21, 22], only 8.2% of the students rated this learning method as ‘most important' with an average of 2.54 (± 0.799) points on the Likert scale. These figures show that, for medical students during orthopaedic instruction, traditional frontal teaching has lost its importance, a fact that can also be seen in the decreasing number of students attending frontal lectures [23]. Other studies show that learning styles do not change during the course of medical school [18].

One of the problems of frontal teaching is that mediated knowledge is communicated only to a small extent on a sustained basis. Another explanation for the decreasing number of lectures may be that frontal teaching is rigid and ignores individual student needs because of the different levels of preknowledge or the different learning strategies. In addition, lectures take place at given times that may not always be convenient [21]. In fact, frontal teaching is competing with asynchronously available content such as Internet research, which can be done at any time [24].

This study shows that students who had already studied another subject before medical school tended to rate the importance of frontal teaching even lower, which may be due to their experiences and the knowledge that missing lectures does not entail any disadvantages.

Wehrwein et al. already showed in 2007 ‘that male and female students have significantly different learning styles' and demanded that it is the responsibility of the instructor to address this diversity of learning styles and develop appropriate learning approaches [7].

Analysing gender-specific differences in this group, female students preferred to highlight text to a significantly higher extent than male students. Other studies have shown that female students significantly prefer reading and writing [7]. This study also showed that female students tended to higher ratings of Internet research and small-group teachings, whereas male students preferred a digital book version at home. Also other authors described gender-dependent differences [25]. Male students are likely to attribute their success in the classroom to external causes, such as teaching, whereas female students generally see their success being directly related to their efforts in the classroom [7].

Last but not least, analysis of the priority list showed not only new media such as online tutorials or online manuscripts in the top-ranking places but also classic books, whereas e-books and audio books ranked last.

As a medical teacher, it is important to understand how to reach all students by understanding how to transfer information using various techniques. This way, teachers help students to be more effective. To be aware of students' learning styles, medical teachers can assist students in determining their needs. As a student, it is very important to adjust study techniques to best fit individual needs [9]. This study shows the different needs and preferences and should create awareness that the method of instruction applied might not always match the preferred style.

### 4.1. Limitations

As in every study involving questionnaires, some limitations need to be mentioned here. In the present study, the collected data were only analysed descriptively. 92.5% (233/252) of the students filled out the questionnaires completely. When interpreting the results to obtain an extreme case estimation, uncertainty of ± 7.5 percentage points regarding the given response distributions must be assumed in the sense of an informal sensitivity analysis.

The survey was done within a collective of students of one medical faculty half way through their years of study. The assessment and priority perspective could theoretically change in the course of further experiences.

Despite these limitations, this study gives an insight into the preferences in learning styles of medical students in orthopaedic education.

## 5. Conclusion

Although new learning possibilities such as Internet research play an important role in modern medical education, traditional methods retain their importance. Due to the increasing number of female medical students, gender-specific differences in personal learning styles should be considered in education.

## Figures and Tables

**Table 1:** Characteristics of the cohort.

**Item**	**n**	%
**Age (mean/range)**	23 (21-33)	(SD ± 3.12)
**Sex ratio (** **f** **e** **m** **a** **l** **e** **: male)**	144 : 89	61.8 : 38.2
**Vocational training before studying (** **y** **e** **s** **: no)**	59 174	25.3 : 74.7
**Worked in another job before (** **y** **e** **s** **: no)**	49 : 184	79.0 : 21.0
**One part of the parents is also a medical doctor (** **y** **e** **s** **: no) **	71 : 161	30.6 : 69.4
**One parent has an academic degree (** **y** **e** **s** **: no)**	61 : 172	26.2 : 73.8
**Both parents work fulltime (** **y** **e** **s** **: no)**	97 : 136	41.6 : 58.4
**Both parents are self-employed (** **y** **e** **s** **: no)**	72 : 161	30.9 : 69.1

## Data Availability

The data used to support the findings of this study are available from the corresponding author upon request.
